# Surgical Timing, Preoperative Oxygenation, and Sex Are Associated with Oxidative Stress and α_1_-Microglobulin Response in Neonatal Open-Heart Surgery

**DOI:** 10.3390/medsci14030408

**Published:** 2026-07-21

**Authors:** Amanda Kristiansson, Alma M. Borgarsdóttir, Magnus Gram, David Ley, Åsa Jungner

**Affiliations:** 1 Department of Clinical Sciences Lund, Pediatrics, Lund University, 221 84 Lund, Sweden; amanda.kristiansson@med.lu.se (A.K.); alma_maggey.borgarsdottir@med.lu.se (A.M.B.); magnus.gram@med.lu.se (M.G.); david.ley@med.lu.se (D.L.); 2 Department of Pediatric Surgery and Neonatal Care, Skåne University Hospital, 222 41 Lund, Sweden; 3 Department of Biomedical Science, Faculty of Health and Society, Biofilms-Research Center for Biointerfaces, Malmö University, 205 06 Malmö, Sweden

**Keywords:** oxidative stress, critical congenital heart defects, open-heart surgery, A1M, cardiopulmonary bypass, infant

## Abstract

**Background**: Neonatal open-heart surgery induces profound oxidative stress, yet its perioperative dynamics remain incompletely characterized. This study quantified urinary 8-hydroxy-2′-deoxyguanosine (8-OHdG) and 8-isoprostane as markers of oxidative damage, and plasma α_1_-microglobulin (A1M) as an endogenous antioxidant, while exploring the influence of pre- and intraoperative factors. **Methods**: In a prospective cohort of 40 term neonates with critical congenital heart defects undergoing open-heart surgery, serial urinary and plasma samples were collected perioperatively. Biomarker concentrations were analyzed using mixed-effects regression models to assess associations with postnatal age, sex, preoperative oxygenation, oxygen surge at bypass initiation, and cell-free hemoglobin in the prime solution. **Results**: Urinary 8-OHdG and 8-isoprostane increased following bypass separation; 8-OHdG remained elevated through postoperative days 0–2, while 8-isoprostane returned toward baseline by day 1. Plasma A1M declined at bypass initiation, recovered to preoperative levels at separation, and rose thereafter. Females exhibited higher A1M concentrations throughout. Longer time to surgery was associated with greater preoperative oxidative stress, and lower preoperative arterial pO_2_ correlated with increased 8-isoprostane at bypass separation. No statistically significant associations were identified between intraoperative variables and biomarker levels, although the study was designed to be a hypothesis generating study and not powered to detect modest intraoperative effects. **Conclusions:** This exploratory study delineates distinct perioperative trajectories of oxidative stress and antioxidant response in neonates undergoing open-heart surgery, with surgical timing, preoperative hypoxemia, and sex emerging as relevant associations. No statistically significant associations were identified between intraoperative variables and biomarker levels; however, the study was hypothesis-generating in design and not powered to detect modest intraoperative effects.

## 1. Introduction

Oxidative stress, defined as an imbalance between the generation and elimination of reactive oxygen species (ROS), is increasingly recognized as a key mediator of cellular and organ injury. In neonatal open-heart surgery, this imbalance has garnered particular interest as a potential contributor to perioperative morbidity [1,2,3,4].

Previous studies suggest that the oxidative stress response is more pronounced in neonates and amplified in more hypoxemic compared with less hypoxemic infants [5,6]. Yet, direct quantification of oxidative stress in a clinical setting remains challenging as many ROS are highly reactive and short-lived [7,8]. Consequently, an estimation of oxidative stress typically relies on quantification of stable oxidized byproducts. Two such urinary biomarkers, 8-hydroxy-2′-deoxyguanosine (8-OHdG) and 8-isoprostane, reflecting oxidation of DNA and lipid peroxidation, respectively, have previously been quantified in infants undergoing cardiac surgery [9].

The neonatal endogenous antioxidant reserve is limited [10,11] and further depleted during cardiopulmonary bypass (CPB), exacerbating the redox imbalance [2,12]. Among endogenous defenses, α_1_-microglobulin (A1M) serves as a potent scavenger of ROS with intra- and extravascular activity. Beyond its antioxidant activity, A1M possesses tissue housekeeping properties with effects on oxidative stress-induced damage in various contexts [13,14]. However, its perioperative kinetics in neonatal open-heart surgery have not been previously described.

The present study aimed to characterize the perioperative oxidative and antioxidant responses during neonatal open-heart surgery with CPB. We quantified oxidative stress through urinary 8-OHdG and 8-isoprostane concentrations and evaluated the endogenous antioxidative response by measuring plasma A1M concentrations. We hypothesized that infants with lower pO_2_ would display an exaggerated oxidative stress response compared to neonates with higher pO_2_, and that exposure to supranormal oxygen tensions and cell-free hemoglobin, both well-characterized drivers of oxidative stress, would intensify the postoperative oxidative stress response.

## 2. Materials and Methods

### 2.1. Study Subjects

Forty term neonates with isolated critical congenital heart defects were included in the study after obtaining informed consent from their legal guardians. Inclusion criteria included gestational weeks at birth ≥ 36 + 0 and no suspected or confirmed syndrome diagnosis. Neonates with multiple malformations were considered ineligible as were neonates with severe medical instability requiring preoperative extracorporeal membrane oxygenation, neonates having undergone cardiopulmonary resuscitation, or neonates diagnosed with hypoxic–ischemic encephalopathy grades 2–3. Clinical characteristics of included study participants are reported in Table 1.

### 2.2. Study Execution

Arterial blood samples for analysis of plasma A1M concentrations were obtained preoperatively on the day of surgery (*n* = 40, denoted pre), at bypass start (*n* = 40, denoted t00), immediately after bypass separation (*n* = 38, denoted post), at admission to the pediatric intensive care unit (PICU) (*n* = 40, denoted pod0), and at postoperative days 1–2 (*n* = 40 and *n* = 40, respectively, denoted pod1–2). Plasma was separated after centrifugation, aliquoted and snap-frozen on dry ice. Samples were stored at −80 °C until analysis. Blood sampling was discontinued when the arterial line was removed. Urinary samples for analysis of 8-OHdG and 8-isoprostane were obtained preoperatively (*n* = 39, denoted pre), immediately after bypass separation (*n* = 27, denoted post), at PICU admission (*n* = 39, denoted pod0), and at postoperative days 1–2 (*n* = 40 and *n* = 38, respectively, denoted pod 1–2). Samples were aliquoted, snap-frozen on dry ice and stored at −80 °C until analysis. Urinary sampling was discontinued when the urinary catheter was removed.

### 2.3. Biomarker Analysis

In plasma, A1M was quantified according to instructions from the manufacturer (Human Alpha 1-Microglobulin ELISA Kit, Aviva Systems Biology, San Diego, CA, USA). Commercially available ELISA kits were used to quantify oxidative stress markers 8-OHdG (Abcam, Cambridge, UK) and 8-isoprostane (Abcam) in urine. For the 8-isoprostane analysis, triphenylphosphine (Merck Life Science, Darmstadt, Germany) was added as recommended by the manufacturer. To normalize urine concentration, creatinine was measured (BioAssay Systems, Hayward, CA, USA) and 8-OHdG and 8-isoprostane are presented as a creatinine ratio. All absorbances were measured in a plate reader (Infinite F50, Tecan, Männedorf, Switzerland) and concentrations were calculated with the GraphPad Prism 10 (Version 10.3.1; GraphPad Software, Boston, MA, USA) software. Raw and summary data of respective biomarker can be found in the Supplementary Material.

### 2.4. Ethical Considerations

The study was approved by the Swedish Ethical Review Authority (protocol code Dnr 2014/479) and executed in accordance with World Medical Association Declaration of Helsinki.

### 2.5. Explanatory Variables

The influence of sex (male/female), birthweight, gestational age at birth, previous balloon atrial septostomy (yes/no), postnatal age at surgery, preoperative preductal pO_2_, and surgical mortality score defined as RACHS-1 category on urinary 8-OHdG and 8-isoprostane and plasma A1M concentrations were evaluated at all sampled timepoints. The influence of biventricular repair (yes/no), exposure to oxygen surge at bypass start defined as Δ-pO_2_ [pO_2_ at bypass start—preoperative pO_2_], exposure to cell-free hemoglobin as determined as cell-free hemoglobin concentrations in blood prime solution, time of cardiopulmonary bypass (min), time of aortic cross-clamp (min), and presence of ultrafiltration (yes/no) was assessed at timepoints from bypass separation and onwards. The influence of volume of transfused plasma (mL/kg) on selected biomarkers was assessed at timepoints from postoperative PICU admission and onwards.

### 2.6. Statistical Considerations

All statistical analyses were performed in R (version 4.6.0, RStudio version 2026.01.2+418) using the lmerTest, emmeans, ggeffects, and ggplot2 packages [15,16,17]. Variables were assessed for normality using the Shapiro–Wilk test and log_2_-transformed prior to analysis where the assumption of normality was violated.

Given the exploratory nature of the study, the influence of pre-specified explanatory variables on biomarker concentrations was examined using linear mixed-effects models with timepoint, the explanatory variable of interest, and their interaction specified as fixed effects, and patient ID included as a random intercept to account for the repeated measures structure of the data. Pairwise comparisons between timepoints were derived from the fitted mixed models using estimated marginal means via the emmeans package. Benjamini–Hochberg correction was applied to the reported findings to control the false discovery rate for multiple comparisons within each biomarker. Where inspection of the fixed effects correlation matrix revealed substantial collinearity between the intercept and a continuous predictor, that predictor was mean-centered prior to model fitting to improve the stability and interpretability of main effect estimates. Marginal predicted values and their confidence intervals were derived from the fitted mixed models using the ggeffects package and used for graphical representation of model estimates.

## 3. Results

### 3.1. Temporal Trajectory of Urinary 8-OHdG, Urinary 8-Isoprostane, and Plasma A1M Concentrations

Plasma sample availability was between 38 and 40 samples for all timepoints. Urinary sample availability was lowest immediately after bypass separation, when samples were available for 27 of 40 infants; at the remaining urinary timepoints, 38–40 samples were available.

**Figure 1:**
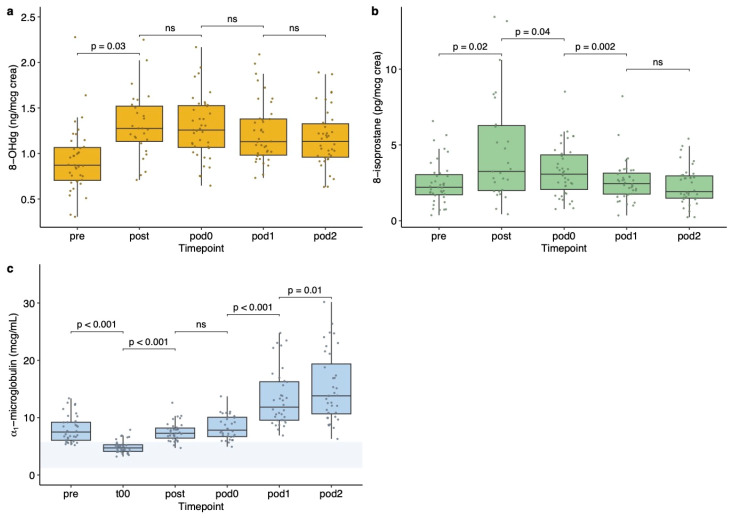
(**a**) Urinary concentrations of 8-hydroxy-2′-deoxyguanosine (8-OHdG) corrected for creatinine. (**b**) Urinary concentrations of 8-isoprostane corrected for creatinine. (**c**) Plasma concentrations of α_1_-microglobulin (A1M). Shaded area represents interquartile range of A1M concentrations in blood prime solution; pre = preoperative, t00 = bypass start, post = bypass separation, pod0 = admission to pediatric intensive care unit (PICU), pod1–2 (postoperative days 1–2). Statistical significance between timepoints were calculated using estimated marginal means derived from mixed linear regression models with Benjamini–Hochberg correction for multiple comparisons.

Urinary 8-OHdG and 8-isoprostane concentrations corrected for creatinine increased significantly immediately after bypass separation compared to preoperative concentrations. Urinary 8-OHdG remained significantly elevated above preoperative levels through postoperative day 2, whereas 8-isoprostane concentrations decreased significantly at PICU admission and returned to levels comparable to preoperative concentrations by postoperative day 1. Plasma concentrations of A1M decreased significantly at bypass start, increased during bypass to levels comparable to preoperative concentrations at bypass separation, and continued to increase thereafter a teach measured timepoint. Individual and aggregated data of urinary 8-OHdG, 8-isoprostane, and plasma A1M are depicted in Figure 1a–c. Descriptive statistics for each biomarker at each timepoint are presented as median and interquartile range in Table 2.

### 3.2. Influence of Demographic and Preoperative Factors on Biomarker Concentrations

#### 3.2.1. Sex and Anthropometric Factors

**Figure 2:**
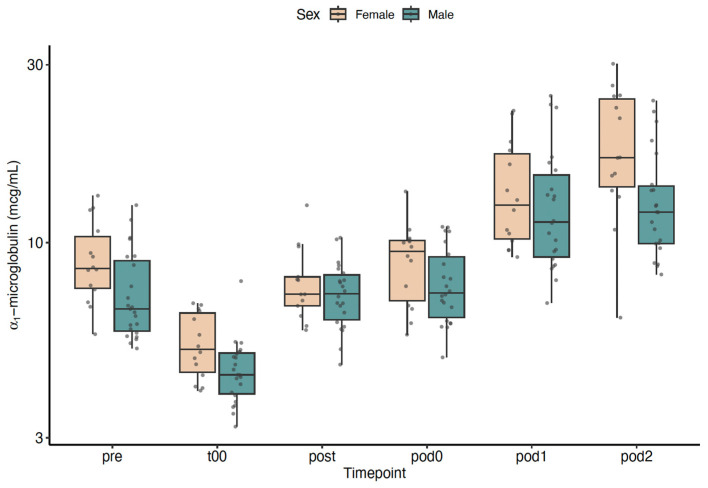
Plasma A1M concentrations stratified according to sex. Note that the *y*-axis is on a log-scale; pre = preoperative, post = bypass separation, pod0 = admission to pediatric intensive care unit (PICU), pod1–2 (postoperative days 1–2). The significant difference between sexes was calculated using mixed linear regression modelling with sex and timepoint as explanatory variables.

Females presented with significantly higher A1M concentrations than males (p adj. = 0.03), Figure 2 and Table 3. However, when a sex-by-timepoint interaction was modelled, the sex difference in plasma A1M was found to vary across the perioperative period, with the divergence between sexes being significantly larger at postoperative day 2 than at any other timepoint (p adj. = 0.01). Sex was not associated with 8-OHdG or 8-isoprostane concentrations at any timepoint. Birthweight and gestational age at birth were not associated with plasma A1M concentrations, urinary 8-OHdG, or urinary 8-isoprostane concentrations at any timepoint.

#### 3.2.2. Postnatal Age at Surgery and Preoperative Oxygenation

**Figure 3:**
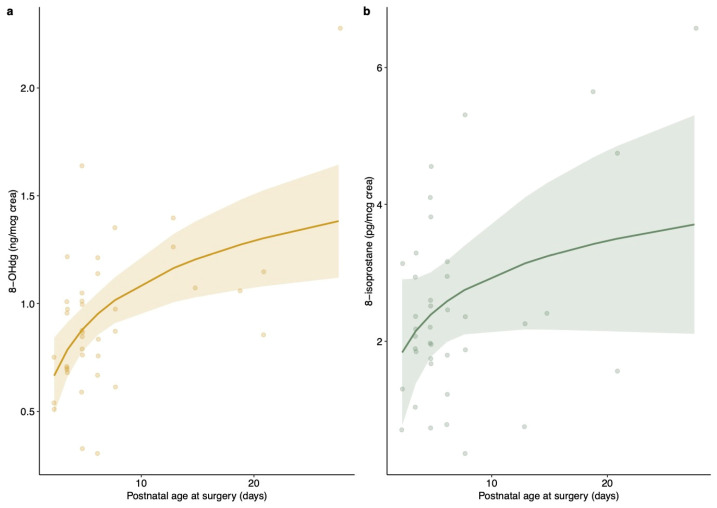
Preoperative urinary concentrations of (**a**) 8-OHdG and (**b**) 8-isoprostane stratified according to postnatal age at surgery displayed as individual data and predicted regression line with shaded area representing 95% confidence intervals around predicted mean.

Increased postnatal age at surgery was significantly associated with higher preoperative urinary concentrations of 8-OHdG (p adj. < 0.001), Figure 3a and Table 3. Increased postnatal age at surgery showed a trend toward higher preoperative 8-isoprostane (p adj. = 0.12), though this did not reach significance in the mixed model, Figure 3b and Table 3. A sensitivity analysis restricted to preoperative observations confirmed a significant positive association between age and preoperative isoprostane levels (p unadj. = 0.013). Paradoxically, the post-bypass surge in both 8-OHdg and 8-isoprostane was significantly attenuated in older infants (p adj. < 0.001 for both), Table 3. These age-dependent effects were confined to the immediate post-bypass timepoint, with no significant interactions observed at later timepoints for either marker.

**Figure 4:**
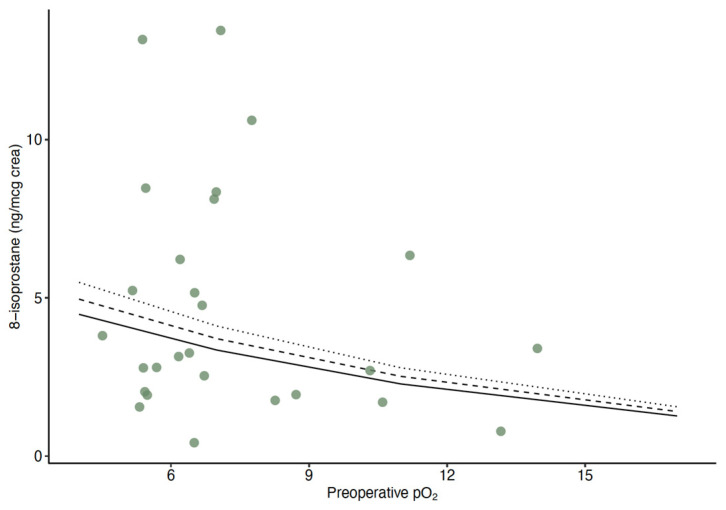
Urinary concentrations of 8-isoprostane after separation from bypass in relation to preoperative pO_2_. The regression lines represent predictive mean concentrations of 8-isoprostane according to the mixed linear regression model where dotted line represents predicted data for postnatal age = 3 days, dashed line predicted data for postnatal age = 6 days and solid line predicted data for postnatal age = 10 days. The number of data points is less than 40 as urinary samples were unobtainable for some patients at bypass separation.

Lower preoperative arterial pO_2_ was associated with increased 8-isoprostane concentrations at bypass separation after adjustment for postnatal age (p adj. = 0.02), Figure 4 and Table 3. This effect was specific to the immediate post-bypass timepoint, with preoperative pO_2_ not significantly associated with urinary 8-isoprostane at any other timepoint, nor with urinary 8-OHdg or plasma A1M concentrations at any timepoint.

Infants having undergone balloon atrial septostomy presented with lower concentrations of 8-OHdG at the preoperative sampling in a multivariable linear regression analysis adjusted for postnatal age at surgery (p adj. = 0.03), Table 3. No effect of prior balloon atrial septostomy was noted for preoperative concentrations of 8-isoprostane and A1M.

### 3.3. Association Between Intraoperative Events and Postoperative Oxidative Stress and Oxidative Stress Response

The magnitude of the hyperoxic exposure at bypass initiation, expressed as Δ-pO_2_ [pO_2_ at t00 − preoperative pO_2_] significantly attenuated the plasma A1M response on postoperative day 1 (p adj. = 0.01), Table 3. Cell-free hemoglobin concentration in the bypass blood prime solution similarily decreased the postoperative day 1 plasma A1M response (p adj. = 0.04), Table 3. Neither Δ-pO_2_ nor prime hemoglobin concentration was significantly associated with plasma A1M at any other timepoint, nor with urinary 8-OHdg or 8-isoprostane concentrations at any timepoint.

Surgical complexity defined by RACHS-1 category, type of repair (biventricular repair or palliative procedure), aortic cross-clamp time, cardiopulmonary bypass duration, fresh frozen plasma transfusion volume (mL/kg), or use of ultrafiltration while on cardiopulmonary bypass (yes/no) were not associated with 8-OHdG-, 8-isoprostane- or A1M concentrations at any of the analyzed timepoints. Similarly, biomarker concentrations at any timepoint were not significantly associated with short-term postoperative outcomes, including acute kidney injury, duration of mechanical ventilation or length of stay in the pediatric intensive care unit.

## 4. Discussion

This article presents data on concentrations of oxidative stress biomarkers 8-OHdG and 8-isoprostane, and the plasma antioxidant A1M during neonatal open-heart surgery. We found that increased postnatal age at surgery was associated with higher preoperative concentrations of 8-OHdG and 8-isoprostane, but that the relative increase in oxidative stress markers during bypass circulation was more pronounced in the youngest. Moreover, we were able to demonstrate that study subjects with lower preoperative pO_2_ presented with an exaggerated intraoperative oxidative stress response during bypass circulation than participants with higher preoperative pO_2_.

Our study subjects present with 10- to 100-fold higher concentrations of urinary 8-OHdG and 8-isoprostane compared to healthy neonates [18,19,20]. We believe that this finding, together with our finding of increased preoperative concentrations of 8-OHdG and 8-isoprostane with a prolonged time to surgery, emphasizes the importance of a preoperative period characterized by varying degrees of unstable hemodynamics and hypoxemia. Our results support previous reports on preoperative perils where increased postnatal age at surgery has been associated with increased incidence of newly acquired postoperative white matter injury and worsened long-term neurological outcome [21,22]. However, the intraoperative oxidative stress response during bypass circulation was more pronounced in younger participants, eliminating the influence of postnatal age at later timepoints. We speculate that the blunted oxidative stress response observed in study subjects operated at a later postnatal age is consistent with a rapid postnatal development of more adept oxidative stress scavenging systems, while also recognizing that our cohort spans a substantively shorter age range than most studies (postnatal age at surgery 3–17 days) [10,11].

We found indications of an exaggerated increase in 8-isoprostane during bypass circulation in the study participants with lower preoperative pO_2_ compared to infants with higher preoperative pO_2_. The correlation between preoperative pO_2_ and the intraoperative increase in 8-isoprostane remained when adjusted for postnatal age. Contrary to our hypothesis, no correlation was found between oxidative stress biomarkers and the pronounced increase in pO_2_ at bypass start, suggesting that a longstanding hypoxemia is more pertinent for the intraoperative oxidative stress reaction than a transitory surge in oxygen tensions at bypass start. We found, however, a correlation between Δ-pO_2_ at bypass start and a blunted response in the antioxidant A1M absolute and relative concentrations at postoperative day 1. We interpret this finding as an increased consumption of A1M following excessive reoxygenation though it must be stressed that no association was noted between Δ-pO_2_ and 8-OHdG, or Δ-pO_2_ and 8-isoprostane concentrations, respectively. No correlation was found between oxidative stress biomarker response and exposure to cell-free hemoglobin which did not support our hypothesis of cell-free hemoglobin as a key contributor of oxidative stress in neonatal open-heart surgery.

Lower preoperative oxidative stress marker levels in infants who had undergone balloon atrial septostomy may reflect improved oxygenation before surgery This interpretation is consistent with previous work suggesting adaptive metabolic buffering of oxidative stress following the procedure [23]. Because balloon atrial septostomy is closely linked to underlying diagnosis and the subgroup size in our cohort was limited, this finding should be interpreted cautiously.

Higher A1M concentrations were observed in female neonates within this exploratory cohort; however, this finding should be interpreted with caution given that females represented only 15 of the 40 neonates included. These elevated concentrations persisted across all time points assessed. No differences between sexes were observed when evaluating 8-OHdG and 8-isoprostane concentrations. Sex-specific oxidative stress and antioxidant response have been reviewed in preclinical and clinical studies where males tend to present an exaggerated oxidative stress reaction and less oxidative stress scavenger resources compared to females [24,25,26]. Notably, sex-specific differences in antioxidant capacity and oxidative stress response have most often been identified in the extravascular compartments, suggesting that accurate characterization of these processes might require other biological samples than plasma.

Antioxidant capacity has been shown to decline during neonatal CPB [2,12]. Circulating A1M concentrations were markedly depleted during bypass circulation, a time of pronounced exposure to various oxidative stressors. The depletion of antioxidants during cardiopulmonary bypass is in line with our previous study in the same cohort where we describe a marked intra- and postoperative decline in concentrations of haptoglobin, a recognized antioxidant and scavenger of cell-free hemoglobin [27].

This study has several limitations. It reflects the practice of a single pediatric cardiac surgery center and lacks clinically relevant long-term outcomes. Urinary samples immediately after bypass separation were available in only 27 of 40 infants, and this missingness was likely non-random: neonates without a sample at this timepoint may have had a compromised renal function, which is in itself associated with greater oxidative stress. Consequently, urinary oxidative stress estimates at this timepoint may underestimate the true oxidative burden. Furthermore, because urinary biomarkers reflect both formation and elimination of oxidative damage products, impaired renal function may have biased estimates even after creatinine normalization. Urinary sampling is also inherently less sensitive than plasma to the marked hemodilution accompanying cardiopulmonary bypass, whereas circulating markers are less dependent on urine output and renal handling. Neither matrix is ideal in isolation; future studies should therefore integrate both urinary and circulating oxidative stress markers alongside dedicated renal function assessment. Finally, the cohort is small, and clinical courses were heterogeneous, though the prospective design and standardized sampling protocol provide a useful foundation for future study design.

Although no statistically significant associations were detected between most intraoperative variables and the measured biomarkers, it must be stressed that the study was not powered to rule out modest intraoperative effects. Previous research has emphasized the difficulties in delineating intraoperative factors influencing postoperative outcome [28,29,30]. However, there is substantial evidence of the deleterious effects of oxidative stress on key maturational events during oligodendrocyte differentiation offering a possible link between cellular biology and the disturbed white matter architecture affecting long-term outcomes in neonates with critical congenital heart defects [31,32]. Moreover, the proposed initiation of immune response and inflammation from oxidative stress is at least hypothesis-generating considering the augmented inflammatory reaction in neonatal open-heart surgery and its proposed association with adverse outcomes [33,34,35]. A thorough understanding of remediable processes during perioperative care is a prerequisite when striving for further improvement in long-term morbidity, but the choice of outcome parameters is fraught with difficulties. We look forward to upcoming antioxidant trials [36].

In conclusion, our findings reveal distinct and time-dependent patterns of oxidative stress and antioxidant response during neonatal open-heart surgery. Urinary markers of oxidative DNA and lipid damage (8-OHdG and 8-isoprostane) displayed rapid alterations, whereas plasma A1M exhibited a delayed increase, suggesting a dynamic compensatory response. Perioperative trajectories of oxidative stress burden and antioxidant response were associated with postnatal age, sex, and preoperative oxygenation. Identifying A1M as a dynamic biomarker of antioxidant capacity and recognizing modifiable factors may help guide strategies to mitigate risk. Larger multicenter studies are warranted to determine whether ameliorating oxidative stress can reduce morbidities in this vulnerable population.

## Figures and Tables

**Table 1:** Clinical characteristics of study cohort. RACHS-1 = Risk Adjustment for Congenital Heart Surgery, ASO = Arterial Switch Operation, VSD = Ventricular Septal Defect, TAPVD = Total Anomalous Pulmonary Venous Drainage, BTT-shunt = Blalock-Thomas-Taussig shunt, CPB = cardiopulmonary bypass, ECMO = extracorporeal membrane oxygenation, PICU = pediatric intensive care unit.

Clinical Characteristics of the Study Cohort	
Sex (male/female) (*n*)	25/15
Birthweight (gr) (median (IQR))	3357 (3234–3592)
Gestational age at birth (weeks) (median (min–max))	39 + 4 (37 + 4–42 + 1)
Ballon atrial septostomy (*n*)	13/40
Postnatal age at surgery (days) (median (IQR))	5 (4–7)
Preductal preoperative pO_2_ (median (IQR))	6.6 (5.6–7.2)
invasive ventilation (*n* = 9)	6.9 (6.8–8.3)
spontaneous ventilation (*n* = 31)	6.2 (5.5–7.0)
RACHS-1 category (*n*)	
3	14/40
4	22/40
6	4/40
Corrective procedure	
ASO + VSD closure (*n*)	11/40
ASO (*n*)	9/40
repair of hypoplastic arch + VSD closure (*n*)	5/40
repair of truncus arteriosus (*n*)	2/40
ASO + repair of hypoplastic arch + VSD closure (*n*)	1/40
repair of hypoplastic arch (*n*)	1/40
repair of TAPVD (*n*)	1/40
Yasui procedure (*n*)	1/40
aortic valvotomy (*n*)	1/40
Palliative procedure	
Norwood procedure (*n*)	3/40
BTT-shunt + atrial septectomy + pulmonary valvotomy (*n*)	2/40
BTT-shunt + atrial septectomy	2/40
BTT-shunt + pulmonary arterioplasty	1/40
Duration of CPB (min) (median (IQR))	182 (142–208)
Duration of aortic cross-clamp (min) (median (IQR))	94 (52–114)
Ultrafiltration (*n*)	23/40
Postoperative ECMO (*n*)	0/40
Postoperative mechanical ventilation (h) (median (IQR))	41 (22–68)
PICU length of stay (h) (median (IQR))	72 (46–122)
Observed 30-day mortality (*n*)	0/40

**Table 2:** Quantification of biomarkers stratified according to timepoint. 8-OHdg = 8-hydroxy-2′-deoxyguanosine, pre = preoperative sampling, t00 = sampling at bypass start, post = sampling after bypass separation, pod0 = sampling at PICU admission, pod1–2 = sampling postoperative day 1–2.

Biomarker	Timepoint					
	pre	t00	post	pod0	pod1	pod2
**8-OHdg (ng/mcg crea)**						
median (IQR)	0.9 (0.7–1.1)	–	1.3 (1.1–1.5)	1.3 (1.1–1.5)	1.1 (1.0–1.4)	1.1 (1.0–1.3)
*n* in final analysis	39	–	27	39	40	38
**8-isoprostane (pg/mcg crea)**						
median (IQR)	2.2 (1.7–3.0)	–	3.3 (2.0–6.3)	3.1 (2.1–4.3)	2.4 (1.8–3.1)	1.9 (1.5–3.0)
*n* in final analysis	39	–	27	39	40	38
**α_1_-microglobulin (mcg/mL)**						
median (IQR)	7.5 (6.1–9.2)	4.7 (4.1–5.3)	7.3 (6.4–8.2)	7.8 (6.7–10.1)	11.8 (9.6–16.3)	13.8 (10.7–16.3)
*n* in final analysis	37	37	35	36	36	36

**Table 3:** Associations between clinical covariates and biomarker levels across perioperative timepoints. β estimates represent the change in biomarker level per unit increase in each predictor, derived from linear mixed models with subject as a random intercept. Predictors marked with ^†^ (postnatal age at surgery, Δ-pO_2_ and cell-free hemoglobin) were log2-transformed prior to analysis; the corresponding β estimates represent the change associated with a doubling of the predictor value. *p*-values were adjusted for multiple comparisons using the Benjamini–Hochberg procedure applied within each biomarker across the three reported covariate effects; 8-OHdg = 8-hydroxy-2′-deoxyguanosine, Δ-pO_2_ = [pO_2_ at bypass start − preoperative pO_2_], Hb = hemoglobin, CPB = cardiopulmonary bypass.

Biomarker and Predictor	Timepoint	β-Estimate	95% CI	p (unadj.)	p (adj.)
**8-OHdg**					
Postnatal age at surgery ^†^	Preoperative	0.29	0.13–0.44	<0.001	<0.001
Ballon atrial septostomy	Preoperative	−0.25	−0.47–−0.03	0.03	0.03
Postnatal age at surgery ^†^	Separation CPB	−0.47	−0.67–−0.28	<0.001	<0.001
					
**8-isoprostane**					
Postnatal age at surgery ^†^	Preoperative	0.75	−0.18–1.67	0.12	0.12
Preoperative pO_2_	Separation CPB	−0.37	−0.66–−0.08	0.02	0.02
Postnatal age at surgery ^†^	Separation CPB	−2.31	−3.36–−1.26	<0.001	<0.001
					
**α_1_-microglobulin**					
Male sex		−1.78	−2.98–−0.32	0.02	0.03
Male sex	Postoperative day 2	−3.46	−5.71–−1.21	0.003	0.01
Δ-pO_2_ ^†^ at start of bypass	Postoperative day 1	−14.7	−25.04–−4.30	0.007	0.01
Exposure to cell-free Hb ^†^	Postoperative day 1	−0.94	−1.80–−0.07	0.04	0.04

## Data Availability

The original contributions presented in this study are included in the article/Supplementary Material. Further inquiries can be directed to the corresponding author.

## Supplementary Materials

The following supporting information can be downloaded at: https://www.mdpi.com/article/10.3390/medsci14030408/s1, Supplementary Material Table S1: 8-hydroxy-2-deoxyguanosine raw and summary data; Supplementary Material Table S2: 8-isoprostane raw and summary data; Supplementary Material Table S3: A1M raw and summary data; Supplementary Material Table S4: Creatinine raw and summary data.

## References

[1] Fudulu D., Angelini G. (2016). Oxidative Stress after Surgery on the Immature Heart. Oxidative Med. Cell. Longev..

[2] Schmidt A.E., Gore E., Henrichs K.F., Conley G., Dorsey C., Bjugstad K.B., Refaai M.A., Blumberg N., Cholette J.M. (2017). Oxidation Reduction Potential (ORP) is Predictive of Complications Following Pediatric Cardiac Surgery. Pediatr. Cardiol..

[3] Kim-Campbell N., Gretchen C., Ritov V.B., Kochanek P.M., Balasubramani G.K., Kenny E., Sharma M., Viegas M., Callaway C., Kagan V.E. (2020). Bioactive Oxylipins in Infants and Children With Congenital Heart Disease Undergoing Pediatric Cardiopulmonary Bypass. Pediatr. Crit. Care Med..

[4] Vázquez D.C., Hadley S.M., Ordóñez M.P., Lopez-abad M., Valls A., Vinals M.L., Moscoso B.A., Benito S., Camprubí-camprubí M., Sanchez-de-Toledo J. (2022). Oxidative Stress and Indicators of Brain Damage Following Pediatric Heart Surgery. Antioxidants.

[5] Sheil M.L., Luxford C., Davies M.J., Peat J.K., Nunn G., Celermajer D.S. (2005). Protein oxidation injury occurs during pediatric cardiopulmonary bypass. J. Thorac. Cardiovasc. Surg..

[6] Vanreusel I., Taeymans J., Van Craenenbroeck E., Segers V.F.M., Van Berendoncks A., Briede J.J., Hens W. (2023). Elevated oxidative stress in patients with congenital heart disease and the effect of cyanosis: A meta-analysis. Free Radic. Res..

[7] Frijhoff J., Winyard P.G., Zarkovic N., Davies S.S., Stocker R., Cheng D., Knight A.R., Taylor E.L., Oettrich J., Ruskovska T. (2015). Clinical Relevance of Biomarkers of Oxidative Stress. Antioxid. Redox Signal..

[8] Murphy M.P., Bayir H., Belousov V., Chang C.J., Davies K.J.A., Davies M.J., Dick T.P., Finkel T., Forman H.J., Janssen-Heininger Y. (2022). Guidelines for measuring reactive oxygen species and oxidative damage in cells and in vivo. Nat. Metab..

[9] Hadley S., Canizo Vazquez D., Lopez Abad M., Congiu S., Lushchencov D., Camprubi Camprubi M., Sanchez-de-Toledo J. (2021). Oxidative stress response in children undergoing cardiac surgery: Utility of the clearance of isoprostanes. PLoS ONE.

[10] Nassi N., Ponziani V., Becatti M., Galvan P., Donzelli G. (2009). Anti-oxidant enzymes and related elements in term and preterm newborns. Pediatr. Int..

[11] Davis J.M., Auten R.L. (2010). Maturation of the antioxidant system and the effects on preterm birth. Pediatr. Semin. Fetal Neonatal Med..

[12] Christen S., Finckh B., Lykkesfeldt J., Gessler P., Frese-Schaper M., Nielsen P., Schmid E.R., Schmitt B. (2005). Oxidative stress precedes peak systemic inflammatory response in pediatric patients undergoing cardiopulmonary bypass operation. Free Radic. Biol. Med..

[13] Bergwik J., Kristiansson A., Allhorn M., Gram M., Akerstrom B. (2021). Structure, Functions, and Physiological Roles of the Lipocalin alpha(1)-Microglobulin (A1M). Front. Physiol..

[14] Burmakin M., Gilmour P.S., Gram M., Shushakova N., Sandoval R.M., Molitoris B.A., Larsson T.E. (2024). Therapeutic alpha-1-microglobulin ameliorates kidney ischemia-reperfusion injury. Am. J. Physiol.-Ren. Physiol..

[15] Kuznetsova A., Brockhoff P.B., Christensen R.H.B. (2017). lmerTest Package: Tests in Linear Mixed Effects Models. J. Stat. Softw..

[16] Lüdecke D. (2018). ggeffects: Tidy Data Frames of Marginal Effects from Regression Models. J. Open Source Softw..

[17] Wickham H. (2016). ggplot2: Elegant Graphics for Data Analysis.

[18] Kaneko K., Kimata T., Tsuji S., Ohashi A., Imai Y., Sudo H., Kitamura N. (2012). Measurement of urinary 8-oxo-7,8-dihydro-2-deoxyguanosine in a novel point-of-care testing device to assess oxidative stress in children. Clin. Chim. Acta.

[19] Shoji H., Oguchi S., Shimizu T., Yamashiro Y. (2003). Effect of human breast milk on urinary 8-hydroxy-2′-deoxyguanosine excretion in infants. Pediatr. Res..

[20] Li S., Hao H., Zhou P., Gao P.M., Xiao X. (2014). Blood and urine 8-iso-PGF2α levels in babies of different gestational ages. Int. J. Clin. Exp. Med..

[21] Lynch J.M., Buckley E.M., Schwab P.J., McCarthy A.L., Winters M.E., Busch D.R., Xiao R., Goff D.A., Nicolson S.C., Montenegro L.M. (2014). Time to surgery and preoperative cerebral hemodynamics predict postoperative white matter injury in neonates with hypoplastic left heart syndrome. J. Thorac. Cardiovasc. Surg..

[22] Lim J.M., Porayette P., Marini D., Chau V., Au-Young S.H., Saini A., Ly L.G., Blaser S., Shroff M., Branson H.M. (2019). Associations between Age at Arterial Switch Operation, Brain Growth, and Development in Infants with Transposition of the Great Arteries. Circulation.

[23] Piñeiro-Ramos J.D., Rahkonen O., Korpioja V., Quintás G., Pihkala J., Pitkänen-Argillander O., Rautiainen P., Andersson S., Kuligowski J., Vento M. (2021). A reductive metabolic switch protects infants with transposition of great arteries undergoing atrial septostomy against oxidative stress. Antioxidants.

[24] Lavoie J.C., Tremblay A. (2018). Sex-Specificity of Oxidative Stress in Newborns Leading to a Personalized Antioxidant Nutritive Strategy. Antioxidants.

[25] Nie X., Lowe D.W., Rollins L.G., Bentzley J., Fraser J.L., Martin R., Singh I., Jenkins D. (2016). Sex-specific effects of N-acetylcysteine in neonatal rats treated with hypothermia after severe hypoxia-ischemia. Neurosci. Res..

[26] Demarest T.G., Schuh R.A., Waddell J., McKenna M.C., Fiskum G. (2016). Sex-dependent mitochondrial respiratory impairment and oxidative stress in a rat model of neonatal hypoxic-ischemic encephalopathy. J. Neurochem..

[27] Jungner Å., Vallius S., Gram M., Ley D. (2022). Cell-Free Hemoglobin Concentration in Blood Prime Solution Is a Major Determinant of Cell-Free Hemoglobin Exposure during Cardiopulmonary Bypass Circulation in the Newborn. J. Clin. Med..

[28] Wernovsky G., Licht D.J. (2016). Neurodevelopmental Outcomes in Children with Congenital Heart Disease-What Can We Impact?. Pediatr. Crit. Care Med..

[29] Morton P.D., Ishibashi N., Jonas R.A. (2017). Neurodevelopmental Abnormalities and Congenital Heart Disease: Insights Into Altered Brain Maturation. Circ. Res..

[30] Bonthrone A.F., Stegeman R., Feldmann M., Claessens N.H.P., Nijman M., Jansen N.J.G., Nijman J., Groenendaal F., de Vries L.S., Benders M. (2022). Risk Factors for Perioperative Brain Lesions in Infants with Congenital Heart Disease: A European Collaboration. Stroke.

[31] Cainelli E., Arrigoni F., Vedovelli L. (2020). White matter injury and neurodevelopmental disabilities: A cross-disease (dis)connection. Prog. Neurobiol..

[32] Ramirez A., Peyvandi S., Cox S., Gano D., Xu D., Tymofiyeva O., McQuillen P.S. (2022). Neonatal brain injury influences structural connectivity and childhood functional outcomes. PLoS ONE.

[33] Gill R., Tsung A., Billiar T. (2010). Linking oxidative stress to inflammation: Toll-like receptors. Free Radic. Biol. Med..

[34] Dietz R.M., Wright C.J. (2018). Oxidative stress diseases unique to the perinatal period: A window into the developing innate immune response. Am. J. Reprod. Immunol..

[35] Prasad J.D., van de Looij Y., Gunn K.C., Ranchhod S.M., White P.B., Berry M.J., Bennet L., Sizonenko S.V., Gunn A.J., Dean J.M. (2021). Long-term coordinated microstructural disruptions of the developing neocortex and subcortical white matter after early postnatal systemic inflammation. Brain Behav. Immun..

[36] Stegeman R., Nijman M., Breur J., Groenendaal F., Haas F., Derks J.B., Nijman J., van Beynum I.M., Taverne Y., Bogers A. (2022). CeRebrUm and CardIac Protection with ALlopurinol in Neonates with Critical Congenital Heart Disease Requiring Cardiac Surgery with Cardiopulmonary Bypass (CRUCIAL): Study protocol of a phase III, randomized, quadruple-blinded, placebo-controlled, Dutch multicenter trial. Trials.

